# Editorial: The Locus of the Stroop Effect

**DOI:** 10.3389/fpsyg.2019.02860

**Published:** 2019-12-17

**Authors:** Benjamin A. Parris, Maria Augustinova, Ludovic Ferrand

**Affiliations:** ^1^Department of Psychology, Bournemouth University, Poole, United Kingdom; ^2^Normandie Université, UNIROUEN, CRFDP, Rouen, France; ^3^Université Clermont Auvergne, CNRS, LAPSCO, Clermont-Ferrand, France

**Keywords:** Stroop, selective attention, cognitive control, task conflicts, semantic conflict, response conflict

One of the famous Monty Python's Holy Grail scenes pictures the Knights of the Round attempting to cross the Bridge of Death. After seeing one of his fellow knights failing to answer a challenging question posed by the Bridge keeper and being cast into the Gorge of Eternal Peril, Sir Galahad nervously approaches the Bridge keeper who asks his name, his quest and….his favorite color. Relieved, he answers with ease before being struck by a sense of dread after saying the color “blue.” The problem, he realizes as he plummets into the gorge, is that his favorite color is in fact yellow…

Even though individuals failing the requirements of the Stroop task (Stroop, 1935) are spared the dread Sir Galahad experienced, they are often heard self-correcting with some sense of consternation: “blue….no yellow! Arghh!”. The Stroop task requires participants to respond quickly to the color a word is printed in whilst at the same time ignoring the meaning of the word itself. The cost of failing to ignore the word is not a plunge into the Gorge of Eternal Peril, but is instead an incorrect response, or more commonly, longer response times compared to when naming the print color of a word that is not color-related (e.g., *club* in yellow). However, almost 30 years after the publication of MacLeod's (1991) seminal review paper, the locus of this so-called Stroop effect remains unclear. The aim of the present Research Topic was to address this still outstanding question.

When aiming to respond to the color, a participant must focus on that task and once perceived, a semantic representation of the color needs to be activated before the associated word form is retrieved. The Stroop effect suggests that this process can be interrupted by the processing of the to-be-ignored word since the task of word reading is seemingly automatically activated, as is the semantic representation of the word and its word form. Potentially then, amongst other loci, the process of color naming could be interrupted at the level of task set activation, semantic processing, and the word form response. Much of the theory and research however has assumed that the interruption, the locus of the Stroop effect, is at the level of responses (Cohen et al., 1990; Roelofs, 2003), and that it is this type of conflict for which control mechanisms monitor (Botvinick et al., 2001). In line with a recent and burgeoning literature (e.g., Parris, 2014; Levin and Tzelgov, 2016; Augustinova et al., 2018; Entel and Tzelgov, 2018; Kalanthroff et al., 2018; Ferrand et al., 2019; Hasshim et al., 2019; Hershman and Henik, 2019; Parris et al., 2019), the contributions to this Research Topic report findings that indicate that there is more than one locus to the Stroop effect.

Littman et al. review the literature on the physiological and behavioral signatures of task conflict and task control in the Stroop task whilst Hsieh and Sharma invoke task conflict and its (proactive) control to account for a general slowdown in color naming of studied non-color neutral and negative emotional words in the Stroop task. Continuing with the notion that emotion can modify Stroop effects, Berger et al. show that age does not affect the use of proactive control over emotion-related Stroop stimuli, but that the nature of the influence of emotion on Stroop effects depends on whether faces or words were the relevant dimension.

The modulating effects of response mode were examined in two contributions. In two experiments, Augustinova et al. report that the locus of interference and facilitation effects might depend on response mode with more types of interference (e.g., task, semantic, and response) and facilitation contributing to the vocal, compared to the manual, response. Highlighting another difference, Mills et al. report a negative priming effect in the vocal (Experiment 1) but not with manual (Experiment 2) Stroop task. The authors argued that this is because it is the actual naming response of the previously ignored stimulus that is suppressed and not the conflict that it generates.

In an fMRI study that controlled for variables that are often confounded, Parris et al. report regions of similar and dissociable neural activity to response and semantic conflict in the Stroop task, whilst Banich summarizes and updates the Cascade-of-Control neural model that argues that there is no single locus to the Stroop effect, and more importantly that the locus might move depending on how well each brain system deals with interference.

In their article, Algom and Chajut argue that the popular “conflict monitoring and control” view of the Stroop effect (Botvinick et al., 2001) fails to account for major Stroop results. Instead, they defend a “data-driven selective attention” view that they argue best accounts for most of Stroop results and one that does not involve higher-order cognitive level processes of control.

Much of the work on the locus of the Stroop effect focusses on the conflict that occurs between the two dimensions of the stimulus on the current trial. However, two studies in this Research Topic build on work showing that interference is smaller on the current trial if the previous trial was incongruent; an effect known as the congruency sequence effect (CSE). Ménétré and Laganaro investigate subprocesses involved in the CSE with participants aged from 10 to 80 years old in order to analyze how interference, CSE, and the decomposition of attention and inhibition change across the lifespan. Aschenbrenner and Balota in contrast examine the relationship of the CSE with another measure of control referred to as the item-specific proportion congruency effect (ISPC).

In sum, these studies, reporting differences at behavioral and neurophysiological levels, highlight the loci of the Stroop effect at the level of task set, semantic, and response selection with the modulating effects of emotion and congruency-sequence.

## Author Contributions

Each author summarized the articles in the Research Topic that they edited. BP wrote the first draft of the editorial. MA and LF provided comments and recommended amendments.

## Conflict of Interest

The authors declare that the research was conducted in the absence of any commercial or financial relationships that could be construed as a potential conflict of interest.
